# Micro-feedback skills workshop impacts perceptions and practices of doctoral faculty

**DOI:** 10.1186/s12909-019-1921-3

**Published:** 2020-01-31

**Authors:** Najma Baseer, James Degnan, Mandy Moffat, Usman Mahboob

**Affiliations:** 1 Institute of Basic Medical Sciences (IBMS), Khyber Medical University, Peshawar, Khyber Pakhtunkhwa Pakistan; 2 Temple University, Philadelphia, USA; 3 Centre For Medical Education, University of Dundee, Dundee, UK; 4 Institute of Health Professions Education & Research, Khyber Medical University, Peshawar, Pakistan

**Keywords:** Postgraduate, Doctoral supervisors, Microteaching, Objective structured teaching exercise (OSTE), Workshop

## Abstract

**Background:**

Doctoral supervision is a distinct form of supervision with clearly defined responsibilities. One of these is the delivery of effective face-to-face feedback to allow supervisees to improve upon their performances. Unfortunately, doctoral supervisors, especially of health sciences, are often not trained in supervisory skills and therefore practice mostly on a trial and error basis. Lack of understanding of the feedback process leads to incongruence in how supervisors and supervisees perceive feedback. However, standardized training practices like microteaching can allow supervisors to acquire effective feedback practices. In this study we employed a schematic approach of microteaching, that is micro-feedback, in a workshop to develop feedback skills of doctoral supervisors, and assessed the overall effectiveness of this training using the Kirkpatrick evaluation framework.

**Methodology:**

This was a Quasi-experimental study with a repeated measures and a two-group separate sample pre-post test design. A micro-feedback skills workshop was organized to enhance feedback skills of doctoral supervisors using microteaching technique. The first two levels of the Kirkpatrick evaluation model were used to determine the workshop’s effectiveness. An informal Objective Structured Teaching Exercise (OSTE) was used to assess feedback skills of the supervisors, both before and after the workshop. A questionnaire was developed to compare pre-and post-workshop perceptions of the supervisors (*n* = 17) and their corresponding supervisees (*n* = 34) regarding the ongoing feedback practice.

**Results:**

Despite the hectic schedule, most doctoral supervisors (17 of 24, 71%) were willing to undertake faculty development training. Participants indicated a high level of satisfaction with the workshop. A learning gain of 56% was observed on pre-post OSTE scores. Prior to the workshop, perceptions of how supervisees should be given the feedback differed significantly between supervisors and supervisees with an effect size difference of *r* = 0.30. After the workshop there was a negligible difference in perceptions between supervisors and supervisees (*r* = .001). Interestingly, supervisors shifted their perceptions more toward those that were originally held by the supervisees.

**Conclusions:**

These findings suggest that well-designed and properly assessed structured programs such as micro-feedback workshops can improve how doctoral supervisors provide feedback to their supervisees and align supervisors’ perceptions of that feedback with those of their supervisees.

## Background

Supervision is the key formal pedagogical method in which the supervisor plays a pivotal role in helping supervisees achieve their learning goals and develop the required professional competence [1, 2]. To use supervision effectively, doctoral supervisors must employ certain distinct skills; in particular, providing timely face-to-face high quality feedback [3–7]. Effective feedback has to be constructive, motivational, comprehensible, and delivered in a timely manner [8]. Feedback given to supervisees not only influences the research process but also deepens supervisees’ understanding of the skills needed to become an effective medical educator [9, 10].

Evidence shows that faculty and students often perceive ongoing feedback practices differently [11]. Supervisors deem supervisees responsible for comprehending and effectively implementing the feedback provided, whereas supervisees are often not content with the quality of feedback they receive and consider it at times inexplicit or confusing [3, 5, 12]. Moreover, medical and allied sciences doctoral supervisors are often not trained in didactic skills, which inhibits them from developing effective supervisory skills and invites them to imitate their own supervisors on a trial and error basis [13, 14]. At the doctoral level, the supervisor-supervisee interactions are mostly based on face-to-face extended systematic conversations or feedback sessions. Moreover, the doctoral supervisors, during their postgraduate experience, are mostly accustomed to the written feedback. The face-to-face supervisory meetings are often unstructured and vary tremendously in terms of frequency and timings. Despite the intricacy of doctoral supervisor-supervisee relationship, no formal training is available to train the doctoral faculty for such interactive sessions.

Multiple feedback models have been developed to facilitate effective feedback practices. The Pendleton feedback model is useful for the feedback process in doctoral PhD supervision interactions and additionally can support inexperienced supervisors to provide specific feedback in a supportive manner [15]. Currently used in many healthcare settings, this model facilitates a two-way interaction between the supervisor and the supervisee, allowing and supporting the supervisees to carry out their own self-assessment.

Various factors influence differences between how supervisors and supervisees perceive the effectiveness of feedback. A lack of training and peer support for supervisors is one [16]. Medical and allied sciences doctoral supervisors are often not specifically trained in supervisory skills, which can inhibit their development in this area and invites them to imitate their own supervisors, often on a trial and error basis [13, 14]. Hence, supervisory training is essential for enhancing the professional development of supervisors.

A paradigm shift in the way medical education is delivered has prompted many faculty development programs to increase the effectiveness of doctoral supervision [17–21]. Some of these programs have employed the method of “microteaching” to develop new supervisory skills and to improve on old ones [22–25]. The term “Micro” symbolizes a more precise and in-depth observation during which special emphasis is given to an explicit pedagogical skill such as effective face-to-face feedback. Thus using the analogy and principles of microteaching, a similar schematic approach of micro-feedback skills can be used to inculcate effective feedback skills among the doctoral faculty. Self-reported perceived improvement in skills acquisition are often unreliable, hence the actual skill level acquired by the supervisors remain to be more robustly evaluated [26]. Direct measures, such as the objective structured teaching exercise (OSTE), can help indicate both a baseline level of a skill and any change that has resulted from a training program [27–29] The direct measure of the evaluation process would allow evaluation of a faculty-training program to go beyond measuring simply ‘Reaction’ to include a more robust measurement of the ‘Learning’ of the skill [30–33].

Keeping in view the acquisition metaphor of learning specially in the context of faculty development [34], this study aimed to assess whether training in a micro-feedback skills workshop leads to an improvement in observed feedback skills of doctoral supervisors individually. Training activities took place in a simulated environment using audiovisual aids and scenarios based on giving immediate feedback to the supervisees [35, 36]. We gauged the effectiveness of the workshop using the first two levels of the Kirkpatrick evaluation model, and we assessed the feedback skills of the doctoral supervisors through micro-training sessions and OSTEs. Lastly, we compared supervisors’ and their corresponding supervisees’ perceptions of the ongoing feedback practices.

## Methods

### Study design & setting

**Fig. 1:**
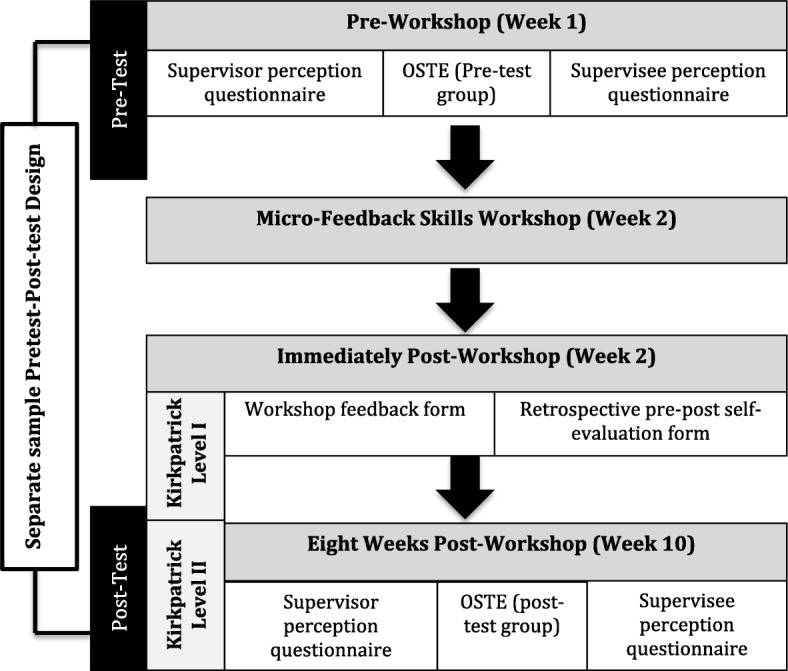
Study Design and data collection procedure. A flowchart showing study design and data collection procedure

This was a quasi-experimental study with two parts; a repeated measures design was used to measure differences in participants’ perceptions of feedback before and after the workshop, and a two-group separate sample pre-post design was used to evaluate the feedback skills of the doctoral supervisors using OSTE [37, 38]. The participants of the workshop, composed of doctoral supervisors belonging to eight different doctoral programs of basic and allied health sciences, were assigned randomly into two groups. One group consisting of half number of participants took part in pre-testing to evaluate their skill level using OSTE, while the other group participated in post-workshop testing, 8 weeks after the workshop (Fig. 1). The setting for this study was Khyber Medical University (KMU) Peshawar.

### Participants

This study targeted doctoral faculty supervisors in different constituent institutes of Khyber Medical University. Twenty-four were invited to participate and 17 consented in writing to take part, three of whom took part in the pilot phase. Participants were then asked to identify two postgraduate supervisees each, who were then invited to participate in the study. Thirty-four supervisees also gave a written consent to take part in the study, six of whom took part in the pilot phase.

### Ethical approval

Ethical approval was obtained from the Graduate Studies Committee, Advanced Studies and Research Board (AS&RB) and the Ethical Committee of Khyber Medical University.

### Pilot testing of the data collection tools

All the instruments and OSTE stations were pilot tested for face validity, content validity, and reliability. The supervisor perception questionnaire was validated using Content Validity Index (CVI) [39, 40] by incorporating data retrieved from seven experienced educationists with teaching and doctoral supervision experience of more than 10 years. Similarly the content was face validated and reliability was computed using the data from three doctoral supervisors and their corresponding six supervisees. OSTE scenarios were pilot tested using standardized students. Two assessors independently established the content validity and inter-rater reliability of the checklist, which were designed based on the principles of Pendleton’s model of effective feedback [15]. The marking rubric for OSTE consisted of two different types of rating scales; a standardized task specific stepwise marking checklist and a global rating scale [41].

### Perception questionnaires

Just before and 8 weeks after the workshop, all participants in the main study completed self-administered 5-point Likert questionnaires (Additional file 1: Annex I and II). The questionnaire was designed following the principles of instrument development [42] and after a thorough literature search [4, 5, 11, 43, 44]. The items in the questionnaires were structured in accordance with the Pendelton’s model of effective feedback to assess the supervisors’ and supervisees’ perceptions of ongoing face-to-face feedback practices [15, 44]. The questionnaire was pilot tested as mentioned above. Changes in participants’ attitudes were analyzed using an approach suggested by Mahmud Zamalia [45]. On a scale of 1 to 5, a score of 2.5 or less was defined as a negative response while a score of 3.5 and above was defined as a positive response. Scores between 2.5 and 3.5 were considered neutral.

### Micro-feedback skills workshop

The workshop was designed keeping in view the acquisition metaphor of learning specially in the context of faculty development [34]. The workshop was interactive in nature and lasted for 8 h. It consisted of four sessions; introductory, behavioral remodeling, micro-feedback skills and workshop feedback sessions. Microteaching was used as an information transfer and training method [22, 23]. The introductory session covered the workshop objectives, the principles of effective feedback, and the elements of microteaching. During the behavioral remodeling session, the participants watched exemplary enactment videos based on the principles of Pendelton’s mode of effective feedback and deliberated on them. During the microteaching session, each workshop participant provided feedback to the trained simulated students [46] based on the pre-determined four doctoral one-to-one feedback scenarios and in return received feedback on their own performance from both the workshop facilitator and the rest of the participants on a microteaching checklist (Additional file 1: Annex III). At the end of the workshop, all the participants were asked to complete a workshop feedback form (Additional file 1: Annex IV) and a pre-post self-evaluation form [47] (Additional file 1: Annex V), both of which corresponded to level I of the Kirkpatrick program evaluation model.

### Objective structured teaching exercise

Before the workshop, each supervisor in the pre-test group participated in an informal OSTE exercise in which the reviewer used a standardized rubric to evaluate the participants’ feedback skills. Designed to accommodate the supervisors’ hectic schedules, this 30-min informal OSTE exercise was conducted in each supervisor’s office setting to accommodate their schedule [28] and to correspond to level II within Kirkpatrick’s program evaluation model. The post-test OSTE exercise took place 8 weeks after the initial exercise, using the same scenarios and checklists as the pre-test [48, 49].

### Data analysis

The data were analyzed using IBM SPSS Statistics version 23. Higher scores on the questionnaire’s Likert scale indicated a more positive rating by the respondents. In addition, effect sizes were computed to measure difference between the supervisors’ and supervisees’ perceptions of the feedback practices. Due to a relatively small sample size, the assumption of underlying normality within the data was evaluated using the Shapiro-Wilk test [50]. For the normally distributed data, independent-sample and paired t-tests were used to compare pre- to post-workshop scores. For non-normally distributed data, the Wilcoxon matched-pair signed-rank test was used for the paired analysis of the matched groups and the Mann-Whitney U test was used to compare two independent groups. Since multiple comparisons were to be made, Bonferroni adjustments to the probability levels needed to indicate statistical significance values were calculated to correct for chance differences. The Bonferroni adjustments for pre- and post-questionnaire item means were .002 (.05/33); and for OSTE items, a value of .002 (.05/28) was required. Based on participants’ pair-wise analyses, the Bonferroni corrected level needed for statistical significance was computed as 0.004 (0.05/14).

## Results

### Pilot testing

After expert validation, the questionnaire was reduced to 33 items and an average Content Validity Index (CVI) of 0.87 was obtained [39, 40]. All 33 items of supervisor questionnaires and 32 items of supervisee questionnaire were rated as relevant and fully understandable with the inter-item reliability index (Cronbach α = 0.79) and (Cronbach α = 0.89), respectively. Similarly, for the OSTE checklists, the coefficient alpha values of 0.79 and 0.86, respectively, for the task-based checklist and for the global rating scale suggested that the checklists were highly reliable.

### Demographics of the participants

A total of 17/24 (71%) of doctoral supervisors (16 male and 1 female) took part in the study. Of these 17, three supervisors took part in the pilot phase of the study while the remaining 14 were included in the main study. Similarly, out of the 34 (14 males and 20 females) corresponding postgraduate supervisees, six took part in the pilot whereas 28 corresponding supervisees participated in the training exercise (Table 1).

**Table 1:** Participants’ demographics in terms of percentages

	Age			Gender		Qualification					Doctoral supervision experience in years			Attended Feedback workshop	
						Doctoral		Basic							
	20–35	36–40	> 40	Male	Female	Basic	Allied	Medicine	Dentistry	Allied	1–3	3–5	> 5	Yes	No
Supervisors (*n* = 17)	42.7	42.9	14.3	92.9	7.1	85.7	14.3	–	–	–	57.1	14.3	28.6	7.1	92.9
Supervisee (*n* = 34)	67.9	25	7.1	42.9	57.1	–	–	53.6	14.3	32.1	–	–	–	–	–

### Evaluation at Kirkpatrick level I

#### Workshop feedback form

The participants rated the workshop very highly, i.e. 4 or higher on a 5-point scale on all 22 items of the workshop feedback form (Table 2). The lowest rating (mean = 3.92, SD = .61) was for the item that asked if the time allotted for the training exercise was sufficient. The maximum rating of 4.93 ± .26 was for the item that asked if the instructor was helpful. In addition, participants were asked to complete two open-ended questions inquiring about the strengths and weaknesses of the workshop. Examples of the comments written in this section included: *“Pendleton’s steps were quite good”, “Proper way of giving feedback to students”, “Well-organized skill enhancement workshop”*, *“Students performed well and near to real life experiences”*, *“Scenarios could have been more diverse”*, *“More scenarios for no repetition”* and *“Four hours were not enough.”*

**Table 2:** Item means of workshop feedback performa

	Workshop Feedback performa items	Mean & SD
1	I was well informed about the objectives of this workshop.	4.12 ± .77
2	This workshop lived up to my expectations.	4.43 ± .51
3	The content is relevant to my needs.	4.79 ± .42
4	The content was organized and easy to follow.	4.64 ± .49
5	The workshop objectives were clear to me.	4.21 ± .69
6	The workshop activities stimulated my learning.	4.35 ± .63
7	The activities in this workshop gave me sufficient practice and feedback.	4.07 ± .73
8	The difficulty level of this workshop was appropriate.	4.00 ± .78
9	The pace of this workshop was appropriate.	4.14 ± .66
10	The method of instruction was appropriate.	4.50 ± .75
11	The meeting room and facilities were adequate.	4.42 ± .75
12	Workshop had a sense of direction.	4.42 ± .51
13	The workshop was a good way for me to learn this content.	4.57 ± .51
14	The time allotted for the training was sufficient. ^a^	3.92 ± .61
15	The instructor was well prepared.^b^	4.93 ± .26
16	The instructor was helpful.	4.85 ± .36
17	Participation and interactions were encouraged.	4.71 ± .46
18	Objectives stated were met.	4.42 ± .51
19	I will be able to use what I learned in this workshop.	4.50 ± .51
20	Overall I will rate the content valuable.	4.42 ± .51
21	I will recommend this workshop to others.	4.57 ± .51
22	I would be interested in attending a follow-up, more advanced workshop on this same subject.	4.85 ± .36

^a^Lowest scoring item

^b^Highest scoring item

### Retrospective pre-post self-evaluation form

For the underlying non-normally distributed data, the Wilcoxon matched-pair signed-rank test was used to assess respondents’ changes in ratings over time. The data showed significant changes in pre- and post-workshop perceptions of all doctoral supervisors (Bonferroni’s correction: sig ≤ 0.004) (Table 3).

**Table 3:** Comparative analysis of pre- and post-workshop self-evaluation form

Participants	Wilcoxon matched-pair signed-rank test (Z values^a^)	Asymp Sig. (2-tailed) **
Participant 1	− 3.051^b^	.002
Participant 2	−3.434 ^b^	.001
Participant 3	−3.873 ^b^	.000
Participant 4	−3.051 ^b^	.002
Participant 5	−3.115 ^b^	.002
Participant 6	−3.145 ^b^	.002
Participant 7	−3.207 ^b^	.001
Participant 8	−2.810 ^b^	.002
Participant 9	−3.068 ^b^	.002
Participant 10	−3.162 ^b^	.002
Participant 11	−3.508 ^b^	.000
Participant 12	−3.443 ^b^	.001
Participant 13	−3.332 ^b^	.001
Participant 14	−3.376 ^b^	.001

**Responses showing significant changes (*p*-value ≤ 0.004)

^a^Statistic value for the test

^b^Based on negative ranks, assigned when the post-test score is higher than the pre-test score and hence their difference gives a negative value

### Evaluation at Kirkpatrick level II

#### Perception questionnaires

The reliability coefficient (α = 0.90) for the pre-workshop perception questionnaire indicated high internal consistency [43]. The following analyses were performed on the data obtained from supervisors and supervisees on the perception questionnaires:

- Pre and post-workshop supervisor perception questionnaires
- Pre and post-workshop supervisee perception questionnaires
- Comparison between supervisor and supervisee perceptions both before and after the workshop.

### Supervisors’ perception questionnaire

Most responses to both the pre- and post-workshop questionnaires showed positive trend with all post-workshop items rated at 3.50 or higher. The highest level of agreement for both pre- and post- workshop questionnaires was found for item no. 1, with mean values of 4.50 ± 0.52 and 4.64 ± 0.50, respectively. Only item no. 6 was rated lower than 3.0 on the pre-test with a mean value of 2.64 ± 1.34. The post-workshop response to this item showed a significant positive shift with a mean value of 4.00 ± 0.78. All other items on the supervisor post-test questionnaire were rated in the positive range (Table 4).

**Table 4:** Pre and post workshop supervisor and supervisees ratings of feedback practice items

Questionnaire Items	Mean values of the items on 5 point Likert scale					
	Supervisor perceptions			Supervisee perceptions		
	Pre workshop	Post workshop	*p*-value	Pre workshop	Post workshop	*p*-value
The supervisor…						
1. Is available for a planned meeting with supervisees	4.5 ± .52	4.6 ±. 50	.435	4.4±. 73	4.5±. 51	.523
2. Selects an appropriate time and place to give feedback	3.8 ± .97	4.1 ± .66	.111	4.6±. 69	4.8 ± .74	.588
3. Informs supervisee if there is a delay in the feedback session	3.9 ± 1.0	3.9 ± .70	.165	4.6±. 63	4.6 ± .62	.839
4. Gives self, enough time to prepare the feedback	3.4 ± .84	3.8 ± .89	.068	-^**^	-^**^	-^**^
5. For feedback on written material, reads the draft prior to the feedback session	4.1 ± .73	4.5 ± .52	.082	4.4±. 79	4.4 ± .74	.861
6. Instructs supervisee to document the proceedings of the feedback session	2.6 ± 1.3	4.0 ± .78	.007	2.7 ± 1.18	3.7 ± .76	.001*
7. Gives supervisee an opportunity to discuss feedback face to face	4.1 ± .53	4.4 ± .65	.002*	4.6±. 74	4.6 ± .73	.857
8. Prepared to handle any complicated situation whilst providing the feedback	4.1 ± .66	4.4 ± .50	.027	3.7±. 91	3.5 ± .96	.693
9. Keeps emotions in check during the feedback session	3.8 ± .43	4.1 ± .61	.165	3.4 ± 1.10	3.4 ± .99	.901
10. Keeps his/her voice in control during the feedback session	4.1 ± .73	4.3 ± .61	.001*	4.6±. 74	4.6 ± .73	.846
11. Tries to make eye contact with supervisee during the feedback session	4.3 ± .61	4.4 ± .65	.002*	4.6±. 69	4.6 ± .69	.857
12. Keeps the feedback process pertinent to the relevant content	3.9 ± .86	4.3 ± .61	.110	4.5±. 79	4.5 ± .79	.861
13. Is capable of making supervisees understand his/her expectations during the feedback session	3.9 ± .73	4.1 ± .36	.336	4.4±. 87	4.5 ± .58	.599
14. Provides specific information about supervisee’s performance	4.0 ± .68	4.0 ± .55	1.00	4.5±. 64	4.5 ± .64	.832
15. Explains the impact of supervisee’s actions on their professional development	3.6 ± 1.0	4.4 ± .65	.001*	4.5±. 79	4.5 ± .79	.846
16. Discusses solutions to problems faced by supervisees during the research	4.1 ± .73	4.4 ± .63	.054	4.3 ±. 85	4.7 ± .44	.548
17. Guides supervisees if they are not performing effectively	3.7 ± .83	4.5 ± .65	.001*	4.3±. 80	4.2 ± .99	.857
18. Helps supervisees acknowledge that a problem exists	4.1 ± .62	4.1 ± .54	.336	4.3 ± 1.02	4.4 ± .99	.802
19. Gives constructive feedback on specific areas to improve upon	4.7 ± .50	4.1 ± .53	.583	4.3 ±. 91	4.4 ± .91	.894
20. Carefully listens to the responses of the supervisee	4.2 ± .70	4.4 ± .76	.139	3.6±. 96	3.7 ± .95	.704
21. Encourages supervisees during the feedback	4.0 ± .68	4.1 ± .66	.068	4.6±. 79	4.6 ± .79	.865
22. Encourages supervisees to probe for more details	3.9 ± .62	4.4 ± .93	.104	4.5±. 80	4.7 ± .74	.839
23. Encourages supervisees to take credit for their success	3.7 ± .61	4.3 ± .73	.007	4.2±. 99	4.4 ± .39	.643
24. Acknowledges the efforts of his/her supervisees	3.9 ± .62	4.4 ± .65	.033	4.5 ±. 29	4.5 ± .79	.832
25. Tries to understand feedback from the supervisee’s viewpoint	3.4 ± .85	4.4 ± .74	.007	4.6±. 57	4.6 ± .57	.832
26. Tries to incorporate the preferred communication style of the supervisee	3.4 ± 1.0	3.6 ± .74	.234	3.4 ± 1.14	3.5 ± 1.07	.000*
27. Attempts to turn every feedback session into a useful encounter	3.4 ± 1.0	3.9 ± .66	.007	4.3±. 89	4.7 ± .82	.754
28. Accepts the responsibility for his/her role in achieving supervisee’s educational goals	3.9 ± .86	4.3 ± .73	.054	4.6±. 88	4.6 ± .21	.876
29. Ensures that the feedback session should be a dialogue, not a monologue	4.1 ± .54	4.4 ± .65	.028	3.1 ± 1.17	3.9 ± 1.09	.826
30. Finishes the feedback session with an action plan for future	3.9 ± .95	4.4 ± .76	.026	4.3±. 85	4.6 ± .23	.765
31. Remembers to appreciate the supervisees after they receive the feedback	3.6 ± .63	4.1 ± .77	.014	3.1 ± 1.04	3.3 ± 1.01	.626
32. Asks supervisees if they have understood the feedback given	3.4 ± .84	4.3 ± .73	.013	4.5±. 88	4.8 ± .92	.921
33. Follows up on his/her previous feedback in the subsequent meeting	3.9 ± .10	4.0 ± .92	.500	3.1 ± 1.18	3.1 ± 1.07	.899

*Responses showing significant shift (*p*-value ≤ 0.002)

**Item was omitted for the supervisee questionnaire as it was only relevant to the supervisors and could not be answered by the supervisees

Paired t-test analysis showed that the supervisor responses to 5 of 33 items (item nos. 7,10,11, 15 and 17) changed significantly after the micro-feedback workshop (*p* ≤ 0.002)(Table 3). Based on the supervisors’ responses, Wilcoxon pair-wise analyses indicated significant shift in the perceptions of more than 50% (i.e. 8/of 14) of the doctoral supervisors (Table 5 Section 1).

**Table 5:** Wilcoxon matched-pair signed-rank test to compare pre- and post test data

Section 1: Comparison of pre- and post-workshop perceptions of the supervisors		
Participant 1	−4.47^a^	.000**
Participant 2	−4.44^a^	.000**
Participant 3	−0.30^a^	.763
Participant 4	−3.27^a^	.001**
Participant 5	− 0.95^a^	.343
Participant 6	−1.80 ^a^	.042
Participant 7	−2.83 ^a^	.005
Participant 8	−2.91 ^a^	.004**
Participant 9	− 2.47 ^a^	.014
Participant 10	−3.67^a^	.000**
Participant 11	−4.17^a^	.000**
Participant 12	−3.66 ^a^	.000**
Participant 13	−4.05 ^a^	.000**
Participant 14	−0.62 ^a^	.536
Section 2: Comparison of pre- and post-workshop perceptions of supervisees regarding the feedback practices of their corresponding supervisors		
Supervisees of Supervisor 1	−0.18^a^	.857
Supervisees of Supervisor 2	−2.84^a^	.002**
Supervisees of Supervisor 3	−1.07^a^	.284
Supervisees of Supervisor 4	−1.92^b^	.055
Supervisees of Supervisor 5	−0.88^a^	.377
Supervisees of Supervisor 6	−0.06^b^	.950
Supervisees of Supervisor 7	−2.18^a^	.029
Supervisees of Supervisor 8	−0.15^b^	.882
Supervisees of Supervisor 9	−0.68^a^	.497
Supervisees of Supervisor10	−1.98^b^	.048
Supervisees of Supervisor11	−2.08^a^	.037
Supervisees of Supervisor 12	−1.21^b^	.226
Supervisees of Supervisor13	−0.69^b^	.494
Supervisees of Supervisor14	−1.31^b^	.192

^a^Based on positive ranks assigned when the pre-test score is higher than the post-test score and their difference gives a positive value

^b^Based on negative ranks, assigned when the post-test score is higher than the pre-test score and hence their difference gives a negative value

**Responses showing significant changes (*p*-value ≤ 0.004)

### Supervisee perception questionnaire

None of the items on the pre-and-post workshop questionnaires were rated negatively (i.e., an average score less than 2.50). The most positive responses to the pre-workshop questionnaire were observed for item no. 2, 3, 7 and 10, 11, 21 and 25 (Table 4), each with an average rating of 4.6, while the most positive response for the post workshop items was for item 2. Pre-post ratings for items 6 and 26 showed significant changes (*p*-value *≤0.002)* (Table 4).

To determine the change in the supervisees’ perceptions regarding the feedback practices of their respective supervisors, the average value of the responses obtained from each supervisor’s two corresponding supervisees were computed. The data were compared using the Wilcoxon matched-pair signed-rank test. The responses obtained from the supervisees of only one supervisor displayed significant change in perceptions of feedback practices (Table 5 section 2).

### Comparison between the perceptions of supervisors and supervisees

The perceptions of supervisors and supervisees towards the ongoing feedback practices differed significantly prior to the workshop. The results of a Mann-Whitney U test comparing the overall pre-test scores to the post-test scores indicated strong differences in perceptions between the two groups (*p* < 0.004) However, the comparison of post-test data indicated that there was no significant difference (*p* = 0.49) between how the supervisors and supervisees perceived feedback practices. The effect size associated with the difference between supervisors’ and supervisees’ average scores on the questionnaire dropped from *r* = 0.29 before the workshop to *r* <.001 after the workshop.

### Objective structured teaching exercise

OSTE sessions were conducted with the supervisors of both pre-test and post-test groups. The OSTE data consisted of checklist-based item scores (performance was scored based on 20 marks) and a global rating scale (5 point scale from 1 to 5), both of which were rated remotely by two raters (Table 6). Results of Shapiro-Wilk tests for data normality confirmed that the pre- and post-workshop checklist data did not violate normality assumptions (*p* = .12 and *p* = .09, respectively). An independent t-test was performed to compare both the pre-test and post-test groups. The OSTE and Global Rating Score post-test gains were significant (t values of − 5.98 (*p* < .001) for the checklist gains and − 4.56 (*p* < .001) for GRS gains. The effect size for the difference associated with the checklist gain was *r* = .51 and for the GRS gain *r* = .60). These score gains indicate that the training program had been effective. Overall learning gains as a result of the workshop were estimated using a procedure recommended by Barwood et al. [51]. Using the Barwood et al. procedure (see formula below), based on the checklist, learning gains of 57% were estimated for the participants in the program.

**Table 6:** OSTE data for both pre-test and post-test groups, showing scores for checklist-based items and global rating scale

Descriptive statistics for OSTE data				
OSTE scores	Minimum score	Maximum score	Mean	Std. Dev
Checklist score for pre-test group (Total score = 20)	6.63	18	13.40	2.81
Checklist score for post-test group (Total score = 20)	13.36	20	17.16	1.75
GRS for pre-test group (Total score = 5)	1.00	5	3.06	1.20
GRS for post-test group (Total score = 5)	2.50	5	4.29	.75

$$ {\displaystyle \begin{array}{c}=\frac{\left( Total\ Post- Test\ Score\ obtained\ast - Total\  Pre- test\ Score\ obtained\ast \ast \right)}{\left( Maximum\ Score\ast \ast \ast - Total\  Pre- test\ Score\ obtained\right)}\times 100\\ {}=\frac{\left(480-375\right)}{560-375}\times 100=56.75\%\end{array}} $$

* Sum of individual checklist scores of all the participants of the post-test group

** Sum of individual checklist scores of all the participants of the pre-test group

*** sum of all the OSTE checklists scores i.e. *20 × 28 = 560*

## Discussion

This study was carried out to determine whether a micro-feedback skills workshop could improve the feedback skills of doctoral supervisors. The findings of this study suggest that a significant improvement was observed not only in the perceived feedback skills of the doctoral supervisors (Kirkpatrick Level 1) but also in observed feedback skills via an OSTE (Kirkpatrick Level 2). Of the 17 supervisors, only one had attended a previous workshop on feedback skills. More generally, very few of the supervisors had participated in workshops designed to improve supervisory skills previous to this study.

### Participants of the study

The participants of this study were a very specific and exclusive group of academicians in the field of basic and allied medical sciences. Although they constituted a relatively small sample, their impact on the supervisees and the ongoing basic medical research is substantial. Pre training, supervisors exhibited high variability in their supervisory practices and there was a marked difference in what they report doing and what their supervisees reported. For example, most supervisors believed that they met informally with their supervisees on a daily basis; yet, supervisees reported that they rarely met with their supervisors on a daily basis.

The second group of participants consisted of the postgraduate research supervisees of the participating doctoral faculty. Handley et al. suggested that an objective assessment of feedback quality is a cumbersome task [43]. Therefore, of all the available tools and resources, students are the best evaluators of the effectiveness of feedback practices. Hence, it was incumbent to incorporate the supervisees’ perceptions of ongoing feedback practices, since they constituted the major stakeholder in this context.

The students varied in terms of their demographics and educational background and these were supervisees who were selected for participation in the study based on their supervisors’ recommendations. Would non-recommended supervisees deviate further from the supervisor’s perceptions? However, for this study we chose to target and invite those more likely to take part, as students are one of the best evaluators of the effectiveness of feedback practices. Moreover, engaging supervisees in workshop activities is important not only for developing their pedagogic literacy but also for understanding the long-term impact of the feedback provided [43].

### The immediate impact of micro-feedback skills workshop

In this study, the levels I and II of the Kirkpatrick program evaluation model (“Reaction” and “Learning”) were fully implemented since these two levels have an individual impact, while levels III and IV have institutional effects [32]. The evaluation of the micro-feedback workshop indicated the faculty perceptions of the importance of feedback (“Reaction”) and their skills in understanding how to deliver feedback (“Learning”) have improved. Moreover, the evidence suggested that some “behavioral modification” did take place and that the supervisors and supervisees were much more likely to agree on the importance of faculty feedback training. The immediate evaluation process (level 1) also highlighted some useful learning points for the workshops for the future. The first is that participants wanted more of it, which was consistent with other similar studies [46, 52, 53]. There were a number of suggestions asking for more variation in the microteaching scenarios used. However, this would require more logistical support and human resources [54]. Participants reported that the videotape interactions and the use of standardized students particularly facilitated the learning process for them. This is similar to participant responses in the Gelula and Yudkowsky study [46].

### The intermediate impact of micro-feedback skills workshop

The intermediate impact of the workshop was gauged using the outcome of perception questionnaires and enhancement in the feedback skills (learning gain) of the workshop participants. The questionnaires completed by the supervisees reflected their evaluation of their supervisors’ feedback skills while the ones completed by their supervisors were a form of self-assessment [11]. The supervisor questionnaire consisted of 33 items and the supervisee questionnaire consisted of 32 items. Except for Item 4 of the supervisor questionnaire, each item of the supervisor questionnaire was also on the supervisee questionnaire. The item omitted on the supervisee questionnaire is relevant to the supervisor and could not be answered by the supervisees. The eight-week gap between pre- and post-assessment lessened the likelihood that the participants remembered the choice they had selected in the pre-workshop questionnaire. Supervisors rated their perceptions of their own training skill abilities highly before the workshop (the average self-rating score exceeded 3.50 on 28 of the 33 items) and at higher levels after the workshop (the average self-rating score exceeded 3.50 on all 33 items). These results indicate that supervisors are considerably confident regarding the feedback they provide. One area of concern relates to the supervisors’ instruction for supervisees to document the proceedings of feedback sessions. Supervisees and supervisors rated this item the lowest on the perception questionnaires. The low rating given to the item about documenting procedures is important because it may explain why poor recall might contribute to the inability of some of the supervisees to follow their supervisors’ instructions and for supervisors to judge whether the supervisees have properly understood the feedback that was given.

The micro-feedback workshop appears to have resulted in significant change in the way supervisors perceive their own feedback practices. Supervisors’ post workshop item means exceeded pre-workshop item means on every item of the supervisors’ questionnaire, and the gains in supervisors’ ratings reached statistically significant levels for 15 of the 32 items. What needs to be explored is whether this change was only for a short term?

Slightly different trends were observed in the data obtained from the supervisee questionnaire. In general, supervisees rated their perceptions of how they received feedback higher than their own supervisors rated how they gave feedback. Supervisees rated their supervisors’ feedback skills higher than their supervisors’ corresponding ratings of themselves on 24 of 32 of the pre-workshop items and on 21 of 32 of the post-workshop items. Supervisees post-workshop averages exceeded pre-workshop averages on 29 of the 32 items, however none of the differences in pre- to post-workshop supervisee item means reached statistically significant levels.

Significant differences were also observed among the pre-workshop perceptions between the supervisors and supervisees; however, no significant differences were found between the two groups after the workshop. The higher post-workshop self-ratings among the supervisors may reflect the supervisors’ improvement in their feedback knowledge and skills but could possibly reflect an attempt to match up to the expectations of their supervisees. The results also imply that in supervisees’ opinion of the feedback practices of their supervisors were satisfactory even before the workshop. The supervisees’ inability to gauge a change in their supervisors’ skills may also be attributed to a lack of pedagogic literacy among the supervisees as they are mostly not involved in faculty development and training programs [43]. A similar finding was also found in a longitudinal study carried out at University of Alberta, in which the students’ ratings of their faculty’s feedback practices were consistent over the period of time of the study [55]. Sidhu et al. [56] compared training programs at different universities and found that they did change supervisees’ perceptions. Their study found high supervisee expectations and highlighted the supervisees’ need for quality feedback from their supervisors. Handley et al. [43] also made similar observations, wherein postgraduate students perceived that their faculty lacked interest in the timely delivery of quality feedback, while the faculty emphasized the quantity of feedback rather than the quality. These conflicting observations can be attributed not only to contextual differences between the study settings but also to the lack of structured faculty development programs and the incoherence of feedback practices at the postgraduate level.

The informal OSTE sessions correspond to the level II (“Learning”) of the Kirkpatrick model of program evaluation and were used to assess the intermediate outcome of the micro-feedback skills workshop by comparing the pre-test and post-test OSTE data. Usually, the pre- and post-test exercise is performed on the same group. However, in this study, a relatively less common separate sample pre- and post-test design was used [38, 57] on different but comparable groups of doctoral supervisors. This was crucial as the available sample was quite small; hence, having a separate control group was not feasible. Also, since the study was skill-based and of short duration, there was a probability of a recall bias and a testing effect. All these issues were effectively dealt with by the use of a separate sample pretest-posttest design [37, 38, 57], which requires a single set of data per participant, and allows generalizability by randomly assigning participants to different observation times [37, 38].

The participants of the study were highly committed faculty members. The availability of all the participants for a traditional multi-station based OSTE was not only logistically challenging but was also overwhelming for the participants. Hence, the OSTE sessions were conducted in a relatively informal but a highly explicit manner, within the office settings of individual supervisors [28]. Unlike in conventional objective structured clinical examinations (OSCE), the participants of this OSTE session were not formally debriefed about their performances. This was not desirable; however, the OSTE used in this study was a faculty development instrument and not an assessment tool. During the workshop, the expert facilitator provided comprehensive feedback about the participant’s performance after each microteaching session. Both a checklist summary score and a global rating score were used to rate the OSTE performance of the participants. Literature suggests that the scores of the global rating scale are more reliable than scores based on item-based checklist scores owing to a more holistic nature of the global rating scale [58]. The combination of both measures in this study resulted in a more comprehensive assessment of participants’ skills The pre-post workshop learning gains on both the global rating and the checklist rating scales were highly significant (more than 56% of learning gain) and the results of the study indicate that the micro-teaching workshops were successful in enhancing the feedback skills of the doctoral supervisors significantly.

### Limitations

This study was not void of limitations. Since the sampling was through census and the participants volunteered for the workshop, the possibility of self-selection bias cannot be eliminated. Therefore, an inherent drive among the participants may have had an impact on the overall level of satisfaction towards the workshop [59]. The sample size was small due to the exceptional nature of the participants; nevertheless, the results indicated statistically significant changes in the perceptions and practices of the participants. The OSTE sessions were videotaped and reviewed remotely by only two reviewers because of the limited number of available experts. Furthermore, the reviewers were not blinded and therefore knew whether the participants belonged to the pre-test or post-test group. However, there was a high consistency in terms of OSTE scores across both the reviewers. The micro-feedback skills workshop was a single-time activity and lacked subsequent reinforcement, which is often desirable to attain long-term learning [59, 60]. Evaluation of the overall impact on the institutions affected by this study (“Results”) will take more time and is outside the scope of this current study. Nonetheless, based on the results of the first two Kirkpatrick evaluation levels, it appears that with circumstantial modifications and a reinforcing element, micro-feedback skills workshops can enhance the feedback skills of postgraduate research supervisors.

## Conclusion

This study assessed the extent to which a micro-feedback skills workshop can influence the feedback practices and perceptions of doctoral supervisors. The workshop was designed to enable supervisors to provide effective feedback to supervisees during training. By assessing the perceptions of supervisors and supervisees pre- and post-workshop, we were able to see their perceptions of the feedback move into alignment with one another. Hence, this study demonstrated that videotaped microteaching and OSTE sessions could be used to enhance supervisory skills. The approach not only provides a more realistic supervisory training experience but also assists in modifying supervisory behaviors and practices. This study also offered a framework for supervisors to develop more effective feedback procedures for use during formal supervisor-supervisee meetings. High-quality feedback that takes place during formal meetings can be very significant for the professional development of both the supervisor and supervisee. The results of this study suggest that faculty development workshops may enhance the knowledge and skills of doctoral medical education faculty as well as faculty involved in other areas of education. More detailed and comprehensive studies are required to establish the relatively long-term effects of micro-teaching training programs at individual and institutional levels.

## Supplementary information

**Additional file 1: **Annexes containing: **Annex I.** Supervisor questionnaire. **Annex II.** Supervisee questionnaire. **Annex III.** Microteaching checklist. **Annex IV.** Workshop Feedback performa. **Annex V.** Workshop pre-post self evaluation form.

## Data Availability

The datasets generated during and/or analysed during the current study are available through the authors and can be made available upon request.

## Abbreviations

BDS: Bachelor of Dentistry
CVI: Content Validity Index
MBBS: Bachelor of Medicine, Bachelor of Surgery
MPhil: Master of Philosophy
OSCE: Objective structured clinical examination
OSTE: Objective structured teaching exercise
PG: Postgraduate
PhD: Doctor of philosophy

## References

[1] Phillips E, Pugh D. How to get a PhD: A handbook for students and their supervisors. McGraw-Hill Education (UK); 2010.

[2] Loganbill C, Hardy E (1983). Developing training programs for clinical supervisors. Clin Superv.

[3] Murphy C, Cornell J (2010). Student perceptions of feedback: seeking a coherent flow. Pract Res High Educ.

[4] Hamid Y, Mahmood S (2010). Understanding constructive feedback: a commitment between teachers and students for academic and professional development. J Pakistan Med Assoc.

[5] Mulliner E, Tucker M (2015). Feedback on feedback practice: perceptions of students and academics. Assess Eval High Educ.

[6] Severinsson E (2015). Rights and responsibilities in research supervision. Nurs Health Sci.

[7] Severinsson E (2012). Research supervision: supervisory style, research-related tasks, importance and quality - part 1. J Nurs Manag.

[8] Mory EH (2004). Feedback research revisited. Handb Res Educ Commun.

[9] Spear R (2000). Supervision of research students: responding to student expectations.

[10] Abiddin NZ (2006). Effective supervision of research students : a study of university practices and foreign students ’ experiences. J Hum Resour Adult Learn.

[11] Ahmed A (2014). Quality of clinical feedback:perceptions of final year BDS students versus their supervisors. J Islam Int Med Coll.

[12] Di Costa N. Feedback on feedback: Student and academic perceptions, expectations and practices within an undergraduate Pharmacy course. InATN Assessment Conference, University of Technology, Sydney, November 2010 (pp. 18-19).

[13] Bitchener J, Basturkmen H, East M (2011). Best practice in supervisor feedback to thesis students.

[14] Delamont S, Atkinson P, Parry O. Supervising the doctorate: a guide to success. 2nd ed: Open University Press; 2005. p. 1–233. Available from: http://eprints.gla.ac.uk/50765/

[15] Chowdhury RR, Kalu G (2004). Learning to give feedback in medical education. Obstet Gynaecol.

[16] Patel P (2016). An evaluation of the current patterns and practices of educational supervision in postgraduate medical education in the UK. Perspect Med Educ.

[17] McAndrew M, Eidtson WH, Pierre GC, Gillespie CC (2012). Creating an objective structured teaching examination to evaluate a dental faculty development program. J Dent Educ.

[18] D’Eon MF (2004). Evaluation of a teaching workshop for residents at the University of Saskatchewan: a pilot study. Acad Med.

[19] Hashim R, Qamar K, Shukr I, Ali S, Khan VA (2014). Faculty perceptions and objective impact of faculty development workshops. Pak Armed Forces Med J.

[20] Smith J (2015). An objective structured teaching exercise ( OSTE ) for physicians employing multi ­ source feedback. MedEdPORTAL Publ.

[21] Pearson M, Brew A (2002). Research training and supervision development. Stud High Educ.

[22] Douglass JE, Pfeiffer IL (1971). Microteaching as a practicum for supervisor education: the effect on supervisor conference behavior and skills.

[23] Douglass JE, Pfeiffer IL (1973). Changes of supervisor behavior in a microteaching practicum. J Exp Educ.

[24] McKnight PC (1971). Microteaching in teacher training: a review of research. Res Educ.

[25] Meier JH (1968). Rationale for and application of microtraining to improve teaching. J Teach Educ.

[26] Kilminster SM, Jolly BC (2000). Effective supervision in clinical practice settings : a literature review. Med Educ.

[27] Stone S, Mazor K, Devaney-O’Neil S, Starr S, Fergusin W, Wellman S (2003). Development and implementation of an objective structured teaching exercise (OSTE) to evaluate improvement in feedback skills following a faculty development workshop. Teach Learn Med.

[28] Boillat M, Bethune C, Ohle E, Razack S, Steinert Y (2012). Twelve tips for using the objective structured teaching exercise for faculty development. Med Teach.

[29] Trowbridge RL, Snydman LK, Skolfield J, Hafler J, Bing-You RG (2011). A systematic review of the use and effectiveness of the objective structured teaching encounter. Med Teach.

[30] Kirkpatrick BD, Kirkpatrick J (2013). Kirkpatrick four levelsš audio recordings study guide.

[31] Parker K, Burrows G, Nash H, Rosenblum ND (2011). Going beyond Kirkpatrick in evaluating a clinician scientist program: itʼs not “if it works” but “how it works”. Acad Med.

[32] Kirkpatrick DL, Kirkpatrick JL (2006). Evaluating training programs (3. 2006).

[33] Yardley S, Dornan T (2012). Kirkpatrick’s levels and education “evidence”. Med Educ.

[34] Sfard A (1998). On two metaphors for learning and the danger of choosing just one. Educ Res.

[35] Barth JL, Shermis SS. Methods of instruction in social studies education. University Press of America; 1984.

[36] Symposium N, Teaching EL (1996). The effects of microteaching supervisory feedback on EFL student teacher performance by Badran A . Hassan, Ph.D. College of Education, Mansoura University.

[37] Campbell DT, Stanley JC (1963). Experimental and Quasi-experimental design for research. Handb Res Teach.

[38] Lynch DC, Johnson JJ (1999). A separate sample pretest-post-test design to evaluate a practice management seminar for residents. Acad Med.

[39] Yaghmale F (2003). Content validity and its estimation. J Med Educ.

[40] Dunning T (2009). Developing and validating a questionnaire to measure spirituality: a psychometric process.

[41] Schol Sandrina (2001). A Multiple-station Test of the Teaching Skills of General Practice Preceptors in Flanders, Belgium. Academic Medicine.

[42] Artino AR, La Rochelle JS, Dezee KJ, Gehlbach H (2014). Developing questionnaires for educational research: AMEE Guide No. 87. Med Teach.

[43] Handley K, Donovan BO, Price M, Millar J (2010). Feedback: all that effort , but what is the effect?. Assess Eval High Educ.

[44] Holmes K, Papageorgiou G (2009). Good, bad and insufficient: students’ expectations, perceptions and uses of feedback. J Hosp Leis Sport Tour Educ.

[45] Mahmud Z (2009). Identification of learners’ attitudes toward statistics based on classification of discriminant function. WSEAS Trans Inf Sci Appl.

[46] Gelula MH, Yudkowsky R (2002). Microteaching and standardized students support faculty development for clinical teaching. Acad Med.

[47] Davis GA (2003). Using a retrospective pre-post questionnaire to determine program impact. J Ext.

[48] Levels TF (2016). Kirkpatrick’s four-level training evaluation model.

[49] Yoon HB, Shin J-S, Bouphavanh K, Kang YM (2016). Evaluation of continuing professional development training program for physicians and physician assistants in hospitals in Laos based on the Kirkpatrick model. J Educ Eval Health Prof.

[50] Ghasemi A, Zahediasl S (2012). Normality tests for statistical analysis: a guide for non-statisticians. Int J Endocrinol Metab.

[51] Barwood CH, Wilson WJ, Malicka AN, McPherson B, Lloyd D, Munt KMB (2013). The effect of rTMS on auditory processing in adults with chronic, bilateral tinnitus: a placebo-controlled pilot study. Brain Stimul.

[52] Kamboj M, Kamboj P, George J, Jha UK (2010). Microteaching in dental education. J Dent Educ.

[53] Hassan BA (1996). The effects of microteaching supervisory feedback on EFL student teacher performance. Proceedings of the 15th national symposium on english language teaching in Egypt.

[54] Remesh A (2014). Microteaching , an efficient technique for learning effective teaching. J Res Med Sci.

[55] Pandachuck K, Harley D, Cook D (2004). Effectiveness of a brief workshop designed to improve teaching performance at the University of Alberta. Acad Med.

[56] Sidhu GK, Kaur S, Fook CY, Yunus FW (2014). Postgraduate supervision: comparing student perspectives from Malaysia and the United Kingdom. Procedia - Soc Behav Sci.

[57] Onwuegbuzie AJ, Witcher AE, Collins KMT, Filer JD, Wiedmaier CD, Moore CW (2007). Students’ perceptions of characteristics of effective college teachers: a validity study of a teaching evaluation form using a mixed-methods analysis. Am Educ Res J.

[58] Siddiqui T, Ahmed A (2013). Reliability of OSTE in the health professions education exit examination of college of physicians and surgeons, Pakistan: a psychometric analysis. J Coll Physicians Surg Pakistan.

[59] Gelula MH, Yudkowsky R (2003). Using standardised students in faculty development workshops to improve clinical teaching skills. Med Educ.

[60] Steinert Y, Mann K, Centeno A, Dolmans D, Spencer J, Gelula M (2006). A systematic review of faculty development initiatives designed to improve teaching effectiveness in medical education: BEME guide no. 8. Med Teach.

