# Supplementary material for: Micro-feedback skills workshop impacts perceptions and practices of doctoral faculty
Source: BMC Med Educ. 2020 Jan 31;20:29. doi: 10.1186/s12909-019-1921-3 (PMC6995071; doi:10.1186/s12909-019-1921-3)
Supplement: Supplementary file 1 — Additional file 1: Annexes containing: Annex I. Supervisor questionnaire. Annex II. Supervisee questionnaire. Annex III. Microteaching checklist. Annex IV. Workshop Feedback performa. Annex V. Workshop pre-post self evaluation form. [file 12909_2019_1921_MOESM1_ESM.docx]

**Supplementary data**

**Micro-Feedback Skills Workshop Impacts Perceptions and Practices of Doctoral Faculty**

Najma Baseer^a^, James Degnan^b^, Usman Mahboob^c,d^

^a^Institute of Basic Medical Sciences (IBMS), Khyber Medical University, Peshawar, Khyber Pakhtunkhwa, ^b^Temple University, Philadelphia, USA ^c^Institute of Health Professions Education & Research, Khyber Medical University, Peshawar, Pakistan, Associate, ^d^Centre for Medical Education, University of Dundee, UK

^a^Dr Najma Baseer MBBS, MHPE, PhD

Assistant Professor in Anatomy

Institute of Basic Medical Sciences (IBMS)

Khyber Medical University

Peshawar, Khyber Pakhtunkhwa

Pakistan
^b^Dr James Degnan PhD

Adjunct Assistant Professor

Senior Director (r) for Measurement and Institutional Research

Temple University, Philadelphia, USA

^c,d^ Dr Usman Mahboob MBBS, MPH, DHPE, FHEA, Fellow FAIMER

Assistant Professor in Medical Education

Institute of Health Professions Education & Research

Khyber Medical University

Peshawar, Pakistan

Associate, Centre for Medical Education
University of Dundee, UK

**Correspondence**

Dr Usman Mahboob

Assistant Professor in Medical Education

Institute of Health Professions Education & Research

Khyber Medical University

Peshawar, Pakistan

Associate, Centre for Medical Education
University of Dundee, UK
Email: [u.mahboob@dundee.ac.uk](mailto:u.mahboob@dundee.ac.uk)

[usman.mahboob@kmu.edu.pk](mailto:usman.mahboob@kmu.edu.pk)
Cell: 0092 333 913 7451

**Annex I: Supervisor perception questionnaire**

**Annex II Supervisee Perception Questionnaire**

**Annex III Microfeedback skills session checklist**

**Annex IV Workshop feedback peforma**

**Annex V Workshop participants’ pre & post self-evaluation form**
